# Enhancing the Predictions of Cytomegalovirus Infection in Severe Ulcerative Colitis Using a Deep Learning Ensemble Model: Development and Validation Study

**DOI:** 10.2196/64987

**Published:** 2025-07-01

**Authors:** Jeong Heon Kim, A Reum Choe, Ju Ran Byeon, Yehyun Park, Eun Mi Song, Seong-Eun Kim, Eui Sun Jeong, Rena Lee, Jin Sung Kim, So Hyun Ahn, Sung Ae Jung

**Affiliations:** 1 Department of Medicine, Yonsei University College of Medicine, Seoul, Republic of Korea; 2 Department of Internal Medicine, College of Medicine, Ewha Womans University Mokdong Hospital, Ewha Womans University College of Medicine, Seoul, South Korea, Seoul, Republic of Korea; 3 Department of Bioengineering, College of Medicine, Ewha Womans University, Seoul, Republic of Korea; 4 Ewha Medical Research Institute, College of Medicine, Ewha Womans University, 25, Magokdong-ro 2-gil, Gangseo-gu, Seoul, Republic of Korea, 82 010-9033-4052

**Keywords:** cytomegalovirus, ulcerative colitis, deep learning, endoscopy, classification

## Abstract

**Background:**

Cytomegalovirus (CMV) reactivation in patients with severe ulcerative colitis (UC) leads to worse outcomes; yet, early detection remains challenging due to the reliance on time-intensive biopsy procedures.

**Objective:**

This study explores the use of deep learning to differentiate CMV from severe UC through endoscopic imaging, offering a potential noninvasive diagnostic tool.

**Methods:**

We analyzed 86 endoscopic images using an ensemble of deep learning models, including DenseNet (Densely Connected Convolutional Network) 121 pretrained on ImageNet. Advanced preprocessing and test-time augmentation (TTA) were applied to optimize model performance. The models were evaluated using metrics such as accuracy, precision, recall, *F*_1_-score, and area under the curve.

**Results:**

The ensemble approach, enhanced by TTA, achieved high performance, with an accuracy of 0.836, precision of 0.850, recall of 0.904, and an *F*_1_-score of 0.875. Models without TTA showed a significant drop in these metrics, emphasizing TTA’s importance in improving classification performance.

**Conclusions:**

This study demonstrates that deep learning models can effectively distinguish CMV from severe UC in endoscopic images, paving the way for early, noninvasive diagnosis and improved patient care.

## Introduction

Cytomegalovirus (CMV) reactivation is frequently observed in patients with severe ulcerative colitis (UC) [1,2]. A multicenter, prospective Korean study found CMV infections in 43% of patients with moderate to severe UC and 67% in those with steroid-refractory UC [3]. Such reactivation often leads to poorer prognoses compared with patients without CMV reactivation, increasing the risk of hospitalization due to UC exacerbation by 8.2 times [4,5]. Antiviral therapy for CMV has been shown to substantially reduce the need for colectomy in patients with severe UC and high-grade CMV infection [6,7]. The American College of Gastroenterology guidelines indicate that CMV superinfection can progress to severe UC, which is resistant to maximum immunosuppressive therapy, necessitating histological analysis through sigmoidoscopy and viral culture for diagnosis [8].

Accurate diagnosis of CMV infection in UC remains critical due to its implications for treatment and prognosis. CMV infection in UC can be diagnosed using blood tests or tissue biopsies. While blood tests are relatively simple and cost-effective, they have limitations in differentiating past or latent infections from active disease. In contrast, tissue biopsies obtained through sigmoidoscopy or colonoscopy provide a more definitive diagnosis but require an invasive procedure. To enhance diagnostic accuracy, targeted biopsies should be taken from both the ulcer base and margins, with McCurdy et al [9] suggesting at least 11 biopsy specimens to achieve over 80% sensitivity. However, collecting multiple specimens increases the risk of bleeding and other complications.

Histopathological techniques used for CMV diagnosis in UC include hematoxylin and eosin (H&E) staining, immunohistochemistry (IHC), and tissue DNA PCR (polymerase chain reaction). H&E staining identifies characteristic cytomegalic cells with nuclear inclusions and has high specificity (92%‐100%) but variable sensitivity (10%‐87%) due to observer dependency. IHC, which detects immediate early antigens of CMV, improves sensitivity (78%‐93%) while maintaining high specificity (92%‐100%). Tissue DNA PCR is the most sensitive (92%‐97%) and specific (93%‐99%) diagnostic tool, but its availability is limited in some institutions, leading to reliance on IHC or H&E staining, potentially delaying diagnosis and treatment [10]. Given the importance of early antiviral intervention in severe UC, diagnostic delays may contribute to adverse clinical outcomes, including increased risk of total colectomy.

Differentiating between severe UC and CMV using endoscopic features presents a challenge [11-13]. Accurate diagnosis of CMV reactivation necessitates techniques like in situ detection of viral markers via specific IHC or nucleic acid amplification [14]. However, these tissue-based diagnostic methods often require several days for results, causing delayed treatment for immunocompromised patients requiring swift intervention. The lag in obtaining tissue biopsy outcomes can pose substantial clinical challenges, notably in CMV reactivation within patients with severe UC. This diagnostic waiting period impedes timely clinical decision-making, potentially exacerbating the patient’s condition. In immunocompromised individuals like those with severe UC, any delay in initiating appropriate therapy can lead to disease progression, heightened morbidity, and even mortality [15,16]. The imperative for prompt intervention underscores the need for more immediate diagnostic approaches.

With the advancement of artificial intelligence (AI), a promising avenue emerges to expedite the diagnosis of CMV reactivation in patients with severe UC [17]. By analyzing complex data patterns and integrating with existing diagnostic methodologies, AI technologies could potentially reduce the time needed to identify CMV reactivation from days to hours. This acceleration in diagnosis would enable health care providers to administer antiviral therapy sooner, potentially improving patient outcomes by reducing the risk of complications and the need for more invasive treatments like colectomy. Furthermore, the application of AI in this diagnostic process could provide a more nuanced understanding of disease progression, facilitating a more tailored approach to treating and managing patients with severe UC and CMV reactivation.

While numerous studies have documented the endoscopic characteristics of CMV infection in UC, none have yet reported on AI systems for distinguishing severe UC from CMV [11-13]. A similar study conducted in 2021 developed a machine learning–based classifier to differentiate CMV from herpes simplex virus esophagitis [18]. This AI system used logistic regression with the least absolute shrinkage and selection operation to discriminate between the 2 conditions, demonstrating 100% sensitivity, specificity, accuracy, and an area under the curve (AUC) of 1.0. The system’s predictive model, using the categorization of endoscopic features and history of transplantation, achieved a high accuracy of 92.6% in distinguishing CMV from herpes simplex esophagitis.

Other viral infections, such as herpes simplex virus, are comparatively rare among patients with UC and do not pose the same level of severe complications and management challenges as CMV reactivation [19-21]. Hence, while it remains imperative to differentiate between CMV and herpes simplex virus infections in patients with UC, accurate diagnosis of CMV infection associated with UC holds heightened clinical significance. This is primarily because CMV has the potential to instigate severe exacerbations of UC and progress to a state of severe UC that proves refractory to standard immunosuppressive therapies [22]. The application of AI technology to swiftly make these distinctions could facilitate the prompt initiation of appropriate interventions, thereby enhancing patient outcomes and mitigating the adverse consequences linked with treatment delays [23].

Numerous studies have reported the remarkable efficacy of AI systems in medical imaging. Various techniques have been proposed to augment and measure ensemble diversity [24]. Recent ensemble studies in medical imaging have demonstrated notable enhancements through multiview approaches, diverse preprocessing methods, and varied network architectures [25,26]. Deep ensemble learning models amalgamate the advantages of deep learning models and ensemble learning to enhance the overall generalization performance of the final model [24]. Consequently, this study aims to address morphological inaccuracies that impede fully automated quantification using deep learning models and to introduce ensemble methods to improve the discrimination of CMV infection in UC.

In the context of endoscopic imaging, predominantly single models have been used. However, discerning CMV infection within UC poses a challenging task, prompting a shift toward various ensemble techniques. Distinguishing these infections through visual inspection demands highly skilled physicians, and even then, the difficulty can vary substantially based on the experience level. This study aims to surpass existing network frameworks by incorporating advanced techniques to enhance the discrimination of CMV infections using deep ensemble learning methods. The rationale behind this approach is to leverage the diverse insights and robustness provided by an ensemble of models, thus overcoming the limitations of single-model approaches and substantially improving the accuracy and reliability of CMV detection in severe UC through endoscopic images [27].

Countless studies have showcased the remarkable prowess of AI systems in medical imaging [28-31]. Techniques for augmenting and measuring ensemble diversity have been put forth, with recent ensemble studies in medical imaging demonstrating notable enhancements via multiview approaches, diverse preprocessing techniques, and varied network architectures [32]. By amalgamating the advantages of both deep learning and ensemble learning, deep ensemble learning models have exhibited improved generalization performance. Thus, this study aims to address morphological errors impeding fully automated quantification using deep learning models and to propose ensemble methods to enhance the discrimination of CMV infection in UC.

## Methods

### Overview

**Figure 1.**
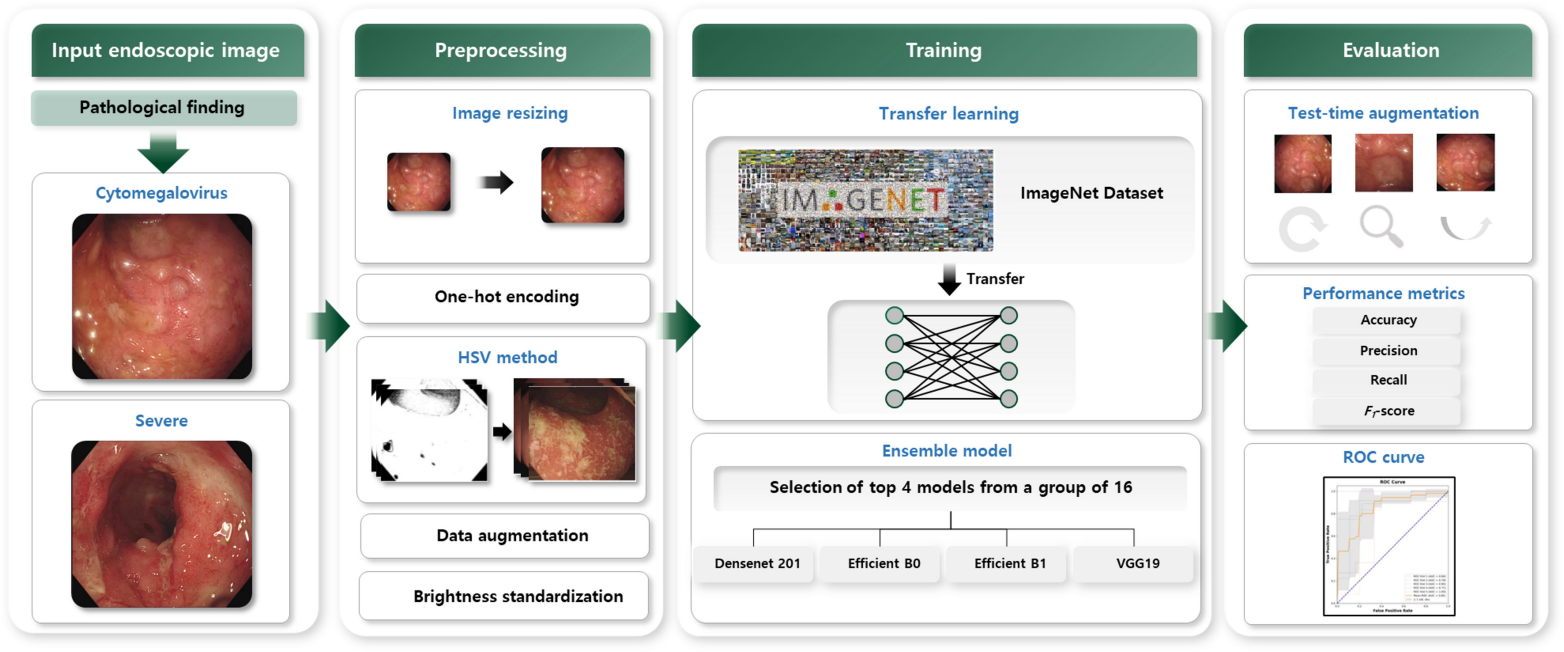
Illustration of the study process: preprocessing endoscopic images of patients with ulcerative colitis with and without cytomegalovirus, applying an ensemble of pretrained models for analysis, using standardization, augmentation, and test-time augmentation to enhance prediction accuracy, and evaluating performance using selected metrics to distinguish between severe ulcerative colitis cases with and without cytomegalovirus complications. HSV: hue, saturation, value; ROC: receiver operating characteristic.

We conducted a thorough analysis of 86 endoscopic images capturing cases of UC, both with and without CMV complications. Before feeding these images into our models, they underwent extensive preprocessing. This phase encompassed standardization for data normalization and augmentation to enhance dataset variability and robustness. We used models pretrained on the extensive ImageNet dataset for the analysis, which is renowned for its diverse image range. Our approach entailed an ensemble of 4 distinct models to leverage their strengths. To further bolster the reliability and accuracy of our predictions, we integrated test-time augmentation (TTA) during the evaluation phase [33]. The effectiveness of this ensemble was evaluated using a range of performance metrics meticulously selected to comprehensively assess the classification performance of our model ensemble, ensuring a comprehensive understanding of its ability to distinguish between cases of with and those without CMV complications (Figure 1).

### Data Collection

We retrospectively reviewed the medical records and endoscopic images of all patients diagnosed with severe UC between February 2019 and December 2022. The severity of UC was assessed using the Mayo Clinic scoring system [34]. The patients included in this study had Mayo Clinic scores ranging from 8 to 12. The presence of CMV was defined as follows: (1) serologic detection of CMV immunoglobin M antibody or (2) histologic detection of inclusion bodies on H&E-stained sections, positive immunohistochemical staining, or CMV DNA amplification by PCR at the first or second evaluation for CMV. Patients were excluded according to the following criteria: coinfection with *Clostridium difficile* and CMV, final pathologic diagnosis of malignancy, or missing information on endoscopic findings. The entire dataset was amassed from a cohort of 34 patients and incorporates endoscopic images sourced from Seoul and Mokdong Hospital of Ewha Womans University Medical Center. Using standardized 8-bit color depth endoscopy systems, specifically the CV-290 (Olympus) at Seoul Hospital, and the CF-Q260AL, CF-H260AL, and CF-HQ290L (Olympus) at Mokdong Hospital, the dataset comprises a total of 86 images (Table 1). This compilation comprises 32 images of CMV cases and 54 images of severe UC, devoid of CMV involvement.

**Table 1.** Dataset information and classification based on histological diagnosis through tissue biopsy.

Index		Severe-UC^a^ without CMV^b^	Severe-UC with CMV
Sex, n			
Male		10	9
Female		11	4
Age (y)			
Mean (range)		51.9 (21-81)	35.4 (12-69)
Median (IQR)		55.5 (41.8-67.2)	34 (25.3-43.6)
Number of images^c^ (CMV, Severe-UC)		54	32

a UC: ulcerative colitis.

b CMV: cytomegalovirus.

c Sampling date: February, 2019-December, 2022.

The resolutions of the dataset were 576×768, 576×720, and 1242×1079 pixels. To ensure the reliability and precision of pathological annotations, each image underwent meticulous analysis based on the consensus findings from tissue biopsies conducted on the patients. Furthermore, 2 experienced pathologists independently reviewed the biopsy results, and their expert opinions were used to annotate each image. In cases where the initial assessments of the pathologists varied, a collaborative review was undertaken to achieve a consensus for the annotations of each image.

### Preprocessing

The endoscopic images were resized to 244×244 pixels to ensure compatibility with transfer learning and standardized for quality across institutions. They underwent hue, saturation, value (HSV) color space transformation, artifact management, data augmentation, pixel value normalization, and one-hot encoding to improve model efficiency and consistency.

### Image Preprocessing and Standardization

To prepare for the application of transfer learning from deep learning models pretrained on the ImageNet dataset, the endoscopic images were resized to a resolution of 244×44 pixels. This resolution is essential to ensure compatibility with the architectures of these pretrained networks, as they require consistent input dimensions.

Given the multi-institutional nature of this research, the endoscopic images were acquired using different endoscopy systems across the participating institutions. To address the variability in image quality parameters, such as brightness and intensity, a meticulous standardization process was implemented for each image. This step proved crucial in normalizing differences resulting from the varied settings and conditions of the endoscopic equipment. Consequently, we ensured systematic adjustment of any discrepancies in image quality, thereby preserving the consistency of the dataset and enhancing the homogeneity of visual information before analysis by the deep learning model. Such standardization facilitates a more reliable comparison and analysis of the images, indispensable for the robust performance of the deep learning model.

### Transformation to HSV Color Space

The images transformed HSV color space to more accurately represent the color distribution and enable effective discrimination between tissue types. Targeted ranges within the HSV color space were defined to standardize the color representation across all images. Furthermore, control mechanisms were implemented to mitigate the influence of artifacts that could affect the model’s performance, such as light reflection and dark areas [35]. To effectively address these artifacts, we established HSV ranges corresponding to them and generated mask images to identify these areas.

### Inpainting Technique

The empty spaces in the mask were filled using an inpainting technique [36]. The inpainting algorithm can follow a model such as:


$$I\left(p\right)=\frac{\sum_{q\in B_{\epsilon }\left(p\right)}w\left(p,q\right)\cdot \left[I\left(q\right)+\nabla I\left(q\right)\cdot \left(p-q\right)\right]}{\sum_{q\in B_{\epsilon }\left(p\right)}w\left(p,q\right)},$$

where *I*(*p*) represents the intensity of the pixel to be inpainted, *I*(*q*) denotes the intensity of a neighboring pixel, and ∇*I*(*q*) signifies the gradient at pixel *q*. The weighting function *w*(*p*,*q*) diminishes with distance, thereby amplifying contributions from closer and more similar pixels, and thereby preserving the natural continuity of biological structures. This approach minimizes errors caused by artifacts and enhances the diagnostic value of the endoscopic images. *I*(*p*) is the color intensity of the point to be inpainted. This equation considers the intensity *I*(*q*) and gradient ∇*I*(*q*) at nearby known points *q*, using a weighting function *w*(*p*,*q*) that accentuates contributions from points directionally aligned with the original image’s isophotes. The weighting function is derived from 3 critical components, directionality, distance, and level set distance, ensuring smooth integration of the inpainting with the surrounding image content. The fast-marching algorithm efficiently processes pixels based on their proximity to the initial boundary, facilitating rapid and consistent restoration of the damaged image areas.

### Data Augmentation

We implemented various data augmentation techniques to enhance the model’s generalization capabilities and replicate real-world variations observed in endoscopic images. These techniques encompassed rotating images across a full 360-degree range to simulate different viewing angles, applying a 15% zoom to simulate variations in image size, shifting the width and height by 20% to introduce positional variations, and shearing by 15% to emulate stress distortions in tissue views. Furthermore, horizontal flipping was used to mitigate orientation biases, and the “reflect” filling mode was used to preserve image integrity during these transformations, ensuring a more comprehensive training process.

**Figure 2.**
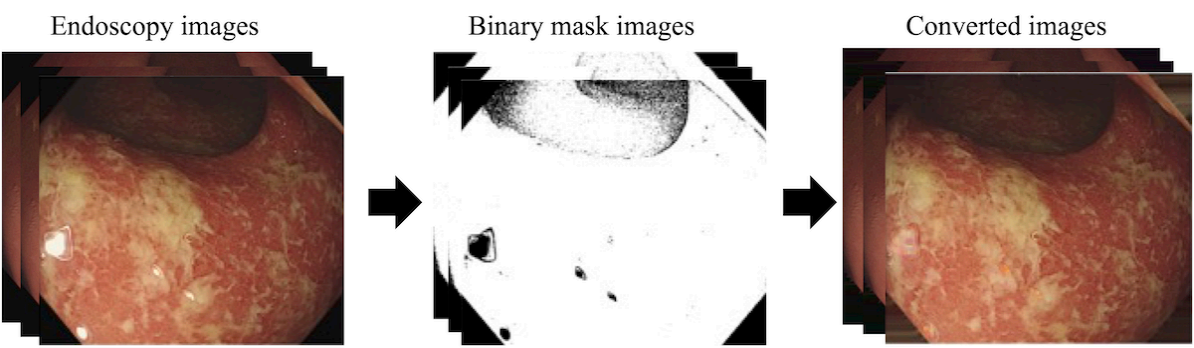
Hue, saturation, and value color space transformation and artifact management in endoscopic images for enhanced diagnostic accuracy.

Pixel values were normalized to a range of (0, 1) to ensure consistency in data input, which is essential for the model’s effective learning. In addition, one-hot encoding was used to convert categorical labels into a binary matrix format, a crucial step for enabling the model to handle binary-class classification tasks efficiently (Figure 2).

### Training Setup

The dataset was divided into training and validation sets in an 8:2 ratio. Input data labeling was based on pathology data, which was considered the gold standard for our classification tasks. Furthermore, 5-fold cross-validation was used to assess single models’ performance within the ensemble. This technique partitions the training dataset into 5 subsets, using each subset iteratively for validation while the remaining data serves as the training set.

### Deep Learning Model

We used a comprehensive ensemble of 16 deep learning models, carefully selected based on their proven effectiveness in image classification tasks, particularly in medical imaging. Each model was constructed using PyTorch (Linux Foundation) and initialized with pretrained weights from the ImageNet dataset. This transfer-learning approach, where a model is pretrained on a large dataset and subsequently fine-tuned for a specific task, is a well-established strategy in medical imaging and has demonstrated substantial efficacy.

Our selection encompassed DenseNet (Densely Connected Convolutional Network), EfficientNet, ResNet, VGG (Visual Geometry Group), Inception, and Vision Transformer architectures, incorporating diverse feature extraction techniques to ensure a comprehensive evaluation of different deep learning methodologies. By leveraging the advanced feature extraction capabilities gained through pretraining on the large-scale and diverse ImageNet dataset, our primary objective was to enhance classification accuracy for endoscopic images. This diversified ensemble approach allowed us to systematically assess each model’s strengths and optimize performance through ensemble learning.

In our study, we implemented patch-based learning to analyze endoscopic images, wherein each of our network models was trained on segmented sections of the images for a more targeted approach. The parameters used in this fine-tuning process are outlined in Table 2, offering a comprehensive overview of the configurations used to optimize our deep learning models for analyzing intricate characteristics of endoscopic images.

**Table 2.** Hyperparameter configurations for fine-tuning deep learning models on endoscopic images.

Hyperparameters	Networks			
	DenseNet^a^ 121	EfficientNet B0	EfficientNet B1	VGG^b^ 19
Initialization	He normal initialization	He normal initialization	He normal initialization	He normal initialization
Batch size	10	10	10	10
Patch size	81×81	81×81	81×81	81×81
Total epochs	10	15	15	15
Optimizer	Adam	Adam	Adam	Adam
Learning rate	0.0001	0.0005	0.0005	0.0001

a DenseNet: Densely Connected Convolutional Network.

b VGG: Visual Geometry Group.

We used He normal initialization and the Adam optimization algorithm to further enhance the performance of our deep learning models. The He normal initialization, also known as Kaiming initialization, was specifically selected for its effectiveness in preserving the variance of the input distributions across layers in deep neural networks, a critical aspect when dealing with complex image data such as endoscopic images. This initialization method addresses the vanishing and exploding gradient issues commonly encountered in training deep architectures, promoting a more stable and efficient optimization process. In addition, we used the Adam optimization algorithm for its adaptive learning rate capabilities, which are crucial for navigating the intricate optimization landscapes inherent in deep learning tasks. Adam combines the benefits of 2 other extensions of stochastic gradient descent—AdaGrad and RMSProp—to adjust the learning rate during training dynamically. This adjustment is based on estimates of lower-order moments of the gradients, facilitating more rapid and effective convergence of our models to the optimal solution.

**Figure 3.**
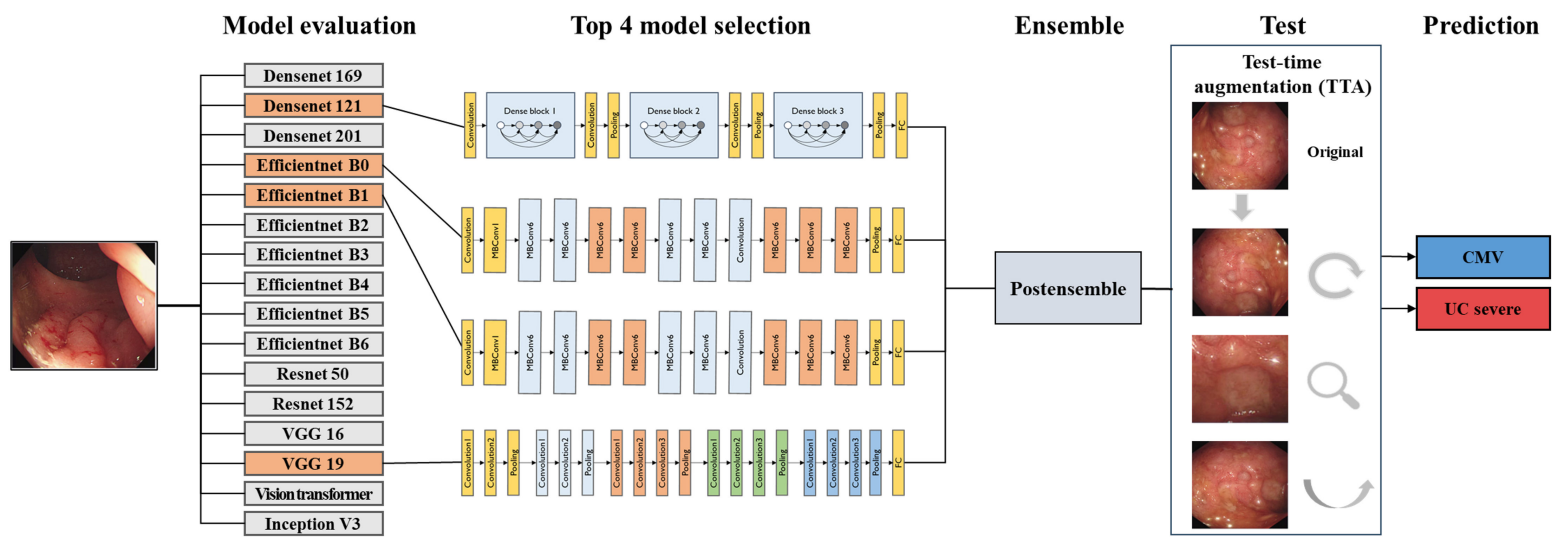
Ensemble deep learning framework for classifying severe ulcerative colitis and cytomegalovirus lesions from endoscopic images. The framework consists of multiple deep learning models pretrained on ImageNet and fine-tuned for the specific classification task. Single model outputs are ensembled to enhance overall classification accuracy. Test-time augmentation, including rotations, zooming, and flipping, is used to ensure robustness against variations in clinical data before making the final prediction. CMV: cytomegalovirus; UC: ulcerative colitis; VGG: Visual Geometry Group.

The top 4 models were selected from a pool of 16 based on their *F*_1_-score, as it is a representative metric that balances precision and recall. In medical image analysis, maintaining a balance between false positives and false negatives is crucial, making the *F*_1_-score a clinically reliable selection criterion. These models were subsequently integrated using a soft-voting ensemble technique, further augmented with TTA to enhance prediction reliability and accuracy (Figure 3). We used an averaging approach to derive the final prediction in the soft-voting procedure. Soft voting was chosen as the optimal ensembling strategy because the models exhibited similar performance, and there was no specific reason to assign higher confidence to any particular model [37,38]. By aggregating the predicted probabilities from multiple models, soft voting provides a more refined decision boundary and yields more stable predictions compared with majority voting. In addition, in the case of weighted voting, adjusting the weights did not result in significant performance improvements over soft voting, and equally incorporating all models through soft voting proved to be the most effective approach. Conversely, TTA involves applying minor variations to the input data during testing, thus providing a more comprehensive evaluation of the model’s performance across diverse conditions. For TTA, we implemented techniques such as horizontal and vertical flips, random adjustments in brightness contrast (±20%), and scaling (30%). These augmentations played a pivotal role in optimizing the predictive capabilities of our system in the challenging realm of endoscopic image analysis, facilitating the introduction of diverse variations and thereby bolstering the model’s ability to generalize and perform consistently across varying imaging scenarios.

In addition, we evaluated ensemble models comprising 2 and 3 models, both with and those without the implementation of TTA. This extensive analysis enabled us to compare the efficacy of varying ensemble sizes and evaluate the impact of TTA on their predictive prowess, specifically emphasizing the averaged soft-voting technique used in tandem with TTA.

### Loss Function

For the binary classification task, the sigmoid activation function was selected for its capability to effectively map input values to a probability distribution ranging from 0 to 1, thereby generating probabilities indicating whether a given input pertains to one class or the other. This made it particularly suitable for discerning between the 2 classes in our study. Complementing this selection, categorical cross-entropy was used as the loss function, given its capacity to quantify the disparity between the predicted distribution and the true distribution of outcomes, which is relevant for binary classification tasks. Categorical cross-entropy measures the difference between the actual label and the predicted probability distribution across classes, thereby furnishing a robust metric for optimizing the model’s performance. By minimizing this loss function, the model is incentivized to adjust its parameters to enhance its predictions’ accuracy, aligning the predicted class probabilities as closely as possible with the ground truth labels.

### Evaluation

We evaluated the performance of ensemble models consisting of 2, 3, and 4 models, both with and without applying TTA. In addition, we assessed the performance of single models.

The assessment used key evaluation metrics, including accuracy, precision, recall, and *F*_1_-score, providing comprehensive insights into each model’s classification performance. In addition, 95% CIs were computed for all evaluation metrics to ensure statistical reliability. The following definitions outline these metrics:


$$Accuracy=\frac{T_{p}+T_{n}}{T_{p}+T_{n}+F_{p}+F_{n}}$$

$$Precision=\frac{T_{p}}{T_{p}+F_{p}}$$

$$Recall=\frac{T_{p}}{T_{p}+T_{n}}$$

$$F_{1}-score=2∙\frac{precision∙recall}{precision+recall}$$

Furthermore, we assessed the diagnostic capabilities of the models through receiver operating characteristic (ROC) curves and their corresponding AUC. These evaluations enabled us to analyze the trade-offs between true positive and false positive rates, as well as to quantify the overall diagnostic accuracy of each model configuration.

To ensure statistical robustness, 95% CIs were computed using 300 bootstrap resampling iterations for all evaluation metrics, including ROC and AUC. This approach enhances the reliability of the performance assessment by providing more stable and generalizable CIs across different evaluation metrics.

### Ethical Considerations

The study was approved by the institutional review board (IRB EUMC 2023-11-009, SEUMC 2023-09-017).

## Results

The performance results of both single models and ensemble model combinations are outlined in Table 3. A comprehensive evaluation of various single models was conducted. DenseNet 121, EfficientNet B0, EfficientNet B1, and VGG 19 stood out for their superior performance, ranking in the top quartile within a cohort of 13 models. Performance metrics, including accuracy, precision, recall, and *F*_1_-score, were recorded along with their 95% CI. DenseNet 121 exhibited exceptional diagnostic reliability for severe UC conditions, achieving an accuracy of 0.792 (95% CI 0.776‐0.808), a precision of 0.821 (95% CI 0.805‐0.837), a recall of 0.876 (95% CI 0.859‐0.893), and an *F*_1_-score of 0.836 (95% CI 0.820‐0.852). EfficientNet B0 demonstrated substantial capability in identifying true positives, with a recall of 0.824 (0.809‐0.839), supported by an accuracy of 0.736 (95% CI 0.720‐0.752), a precision of 0.765 (95% CI 0.746‐0.784), and an *F*_1_-score of 0.784 (95% CI 0.768‐0.800). EfficientNet B1 contributed to reducing false positives, achieving a precision of 0.759 (95% CI 0.742‐0.776), an accuracy of 0.681 (95% CI 0.660‐0.702), a recall of 0.709 (95% CI 0.692‐0.726), and an *F*_1_-score of 0.738 (95% CI 0.720‐0.756). Despite a lower accuracy of 0.660 (95% CI 0.635‐0.685), VGG 19 played a key role in positive case identification, with a precision of 0.707 (95% CI 0.690‐0.724), a recall of 0.745 (95% CI 0.729‐0.761), and an *F*_1_-score of 0.729 (95% CI 0.710‐0.748).

**Table 3.** Average performance metrics with confidence intervals of the top 4 single models and test-time augmentation ensemble models.

Model	Accuracy (95% CI)	Precision (95% CI)	Recall (95% CI)	*F*_1_-score (95% CI)
DenseNet^a^ 121	0.792 (0.776‐0.808)	0.821 (0.805‐0.837)	0.876 (0.859‐0.893)	0.836 (0.820‐0.852)
EfficientNet B0	0.736 (0.720‐0.752)	0.765 (0.746‐0.784)	0.824 (0.809‐0.839)	0.784 (0.768‐0.800)
EfficientNet B1	0.681 (0.660‐0.702)	0.759 (0.742‐0.776)	0.709 (0.692‐0.726)	0.738 (0.720‐0.756)
VGG^b^ 19	0.660 (0.635‐0.685)	0.707 (0.690‐0.724)	0.745 (0.729‐0.761)	0.729 (0.710‐0.748)
Ensemble 4 models with TTA^c^	0.836 (0.824‐0.848)	0.850 (0.832‐0.868)	0.904 (0.890‐0.918)	0.875 (0.860‐0.890)
Ensemble 3 models with TTA	0.723 (0.705‐0.741)	0.814 (0.801‐0.828)	0.789 (0.775‐0.803)	0.802 (0.787‐0.817)
Ensemble 2 models with TTA	0.715 (0.697‐0.733)	0.781 (0.765‐0.797)	0.832 (0.818‐0.846)	0.794 (0.778‐0.810)
Ensemble 4 models without TTA	0.709 (0.692‐0.726)	0.691 (0.675‐0.707)	0.716 (0.700‐0.732)	0.752 (0.737‐0.767)
Ensemble 3 models without TTA	0.775 (0.762‐0.788)	0.753 (0.740‐0.766)	0.759 (0.746‐0.772)	0.755 (0.742‐0.768)
Ensemble 2 models without TTA	0.673 (0.655‐0.691)	0.750 (0.738‐0.762)	0.776 (0.762‐0.790)	0.763 (0.745‐0.781)

a DenseNet: Densely Connected Convolutional Network.

b VGG: Visual Geometry Group.

c TTA: test-time augmentation.

**Figure 4.**
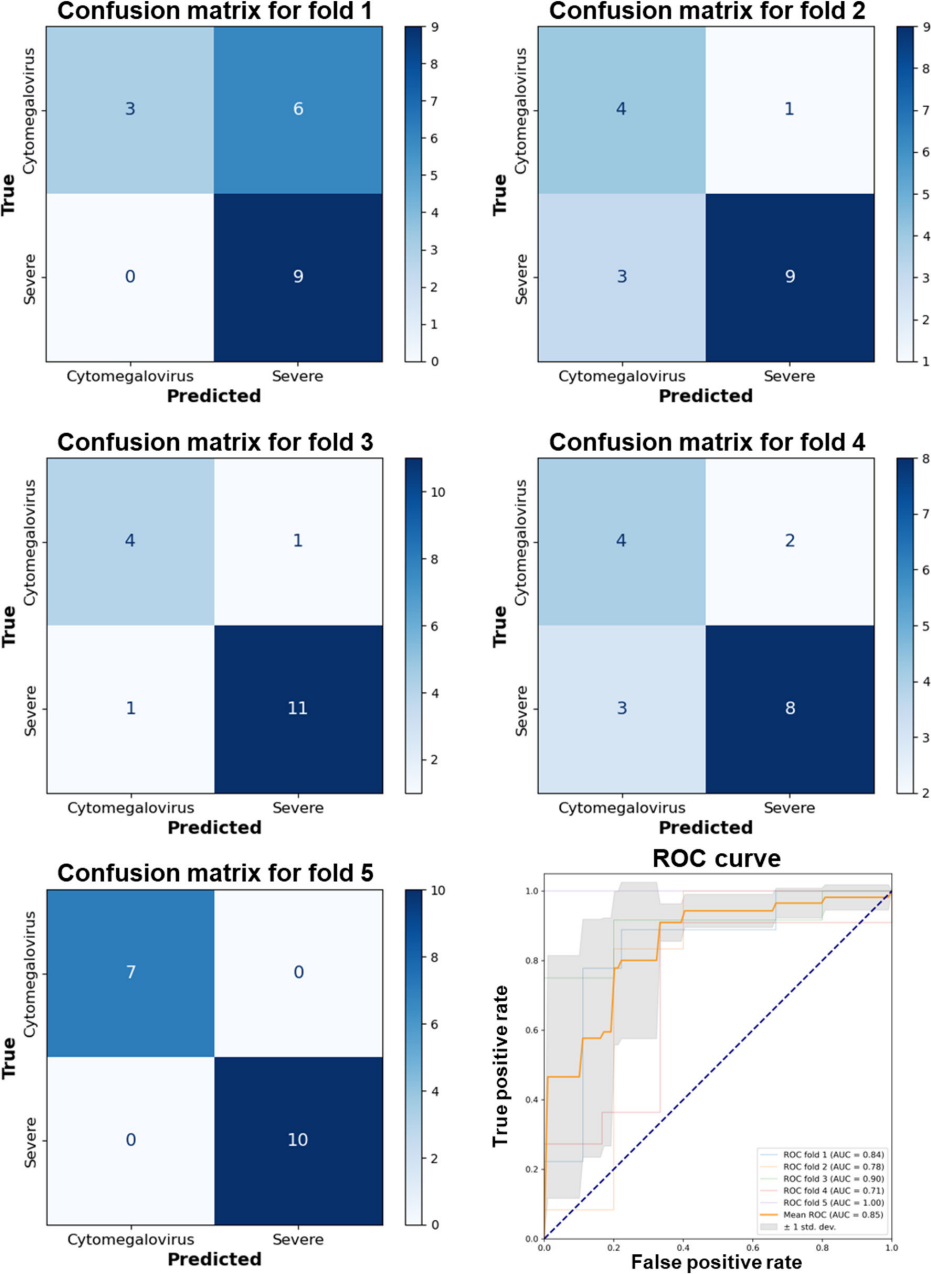
Performance evaluation of the DenseNet 121 model using 5-fold cross-validation and receiver operating characteristic curve analysis. AUC: area under the curve; ROC: receiver operating characteristic.

**Figure 5.**
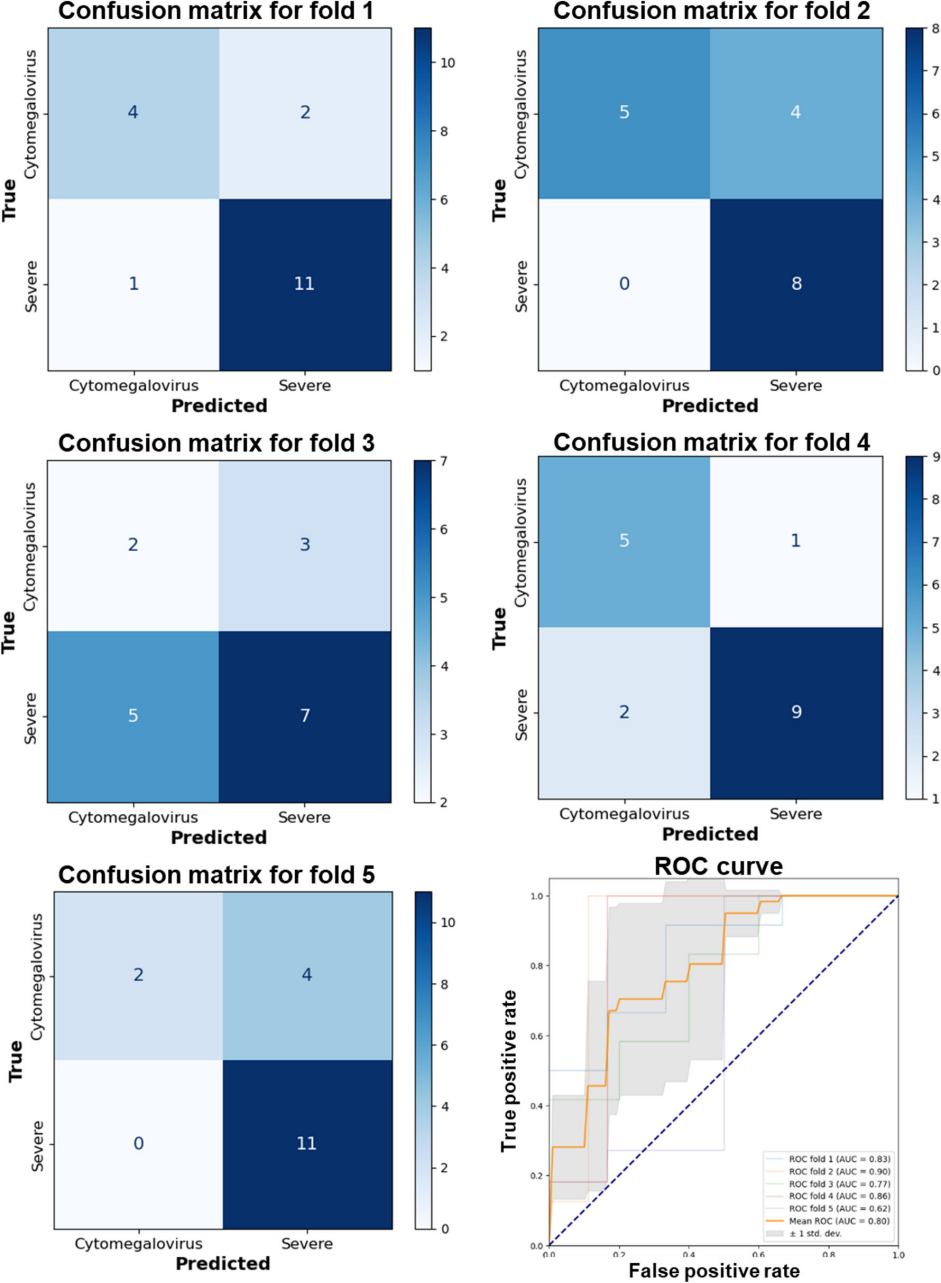
Performance evaluation of the EfficientNet B0 model using 5-fold cross-validation and receiver operating characteristic curve analysis. AUC: area under the curve; ROC: receiver operating characteristic.

**Figure 6.**
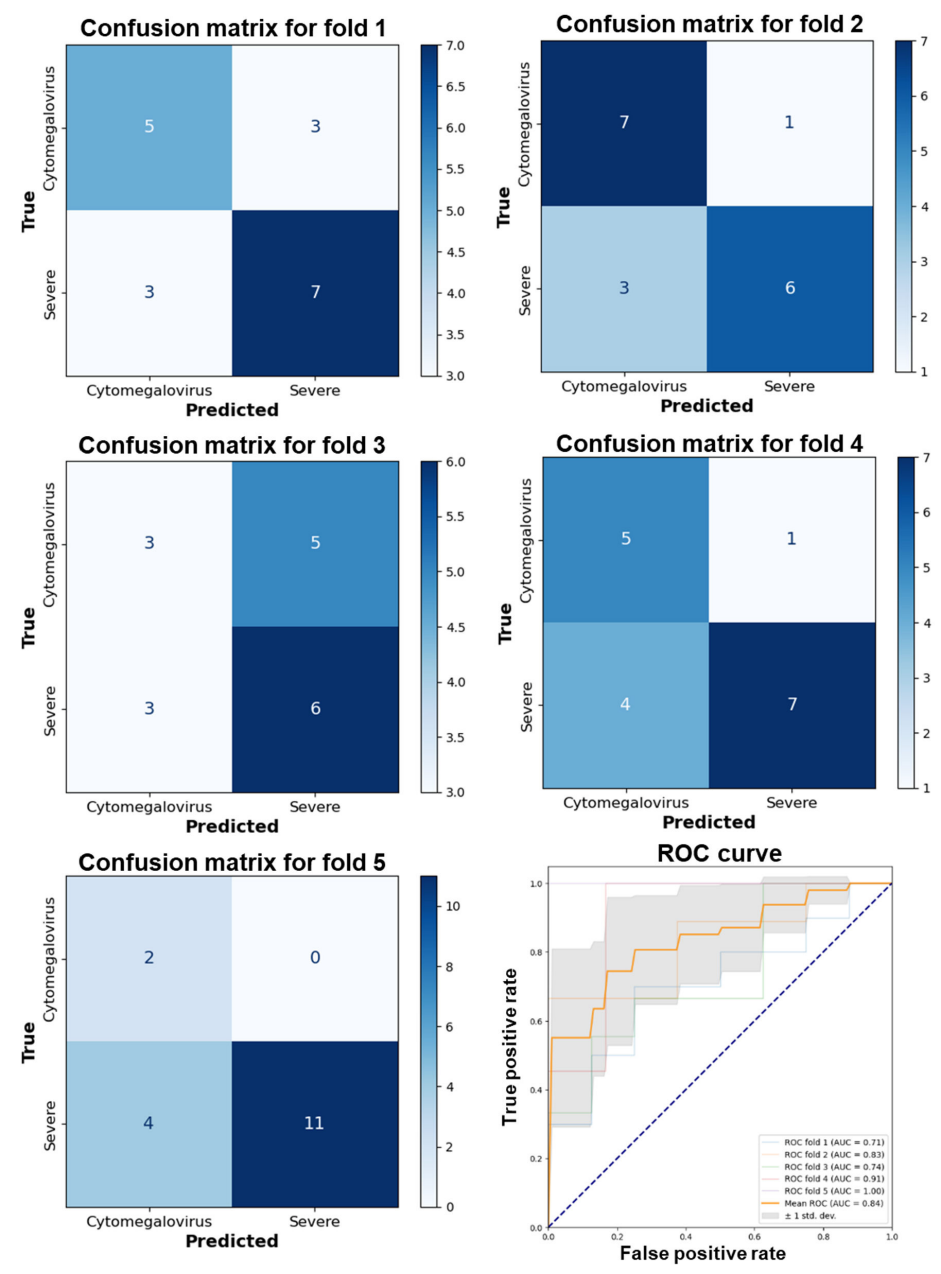
Performance evaluation of the EfficientNet B1 model using 5-fold cross-validation and receiver operating characteristic curve analysis. AUC: area under the curve; ROC: receiver operating characteristic.

**Figure 7.**
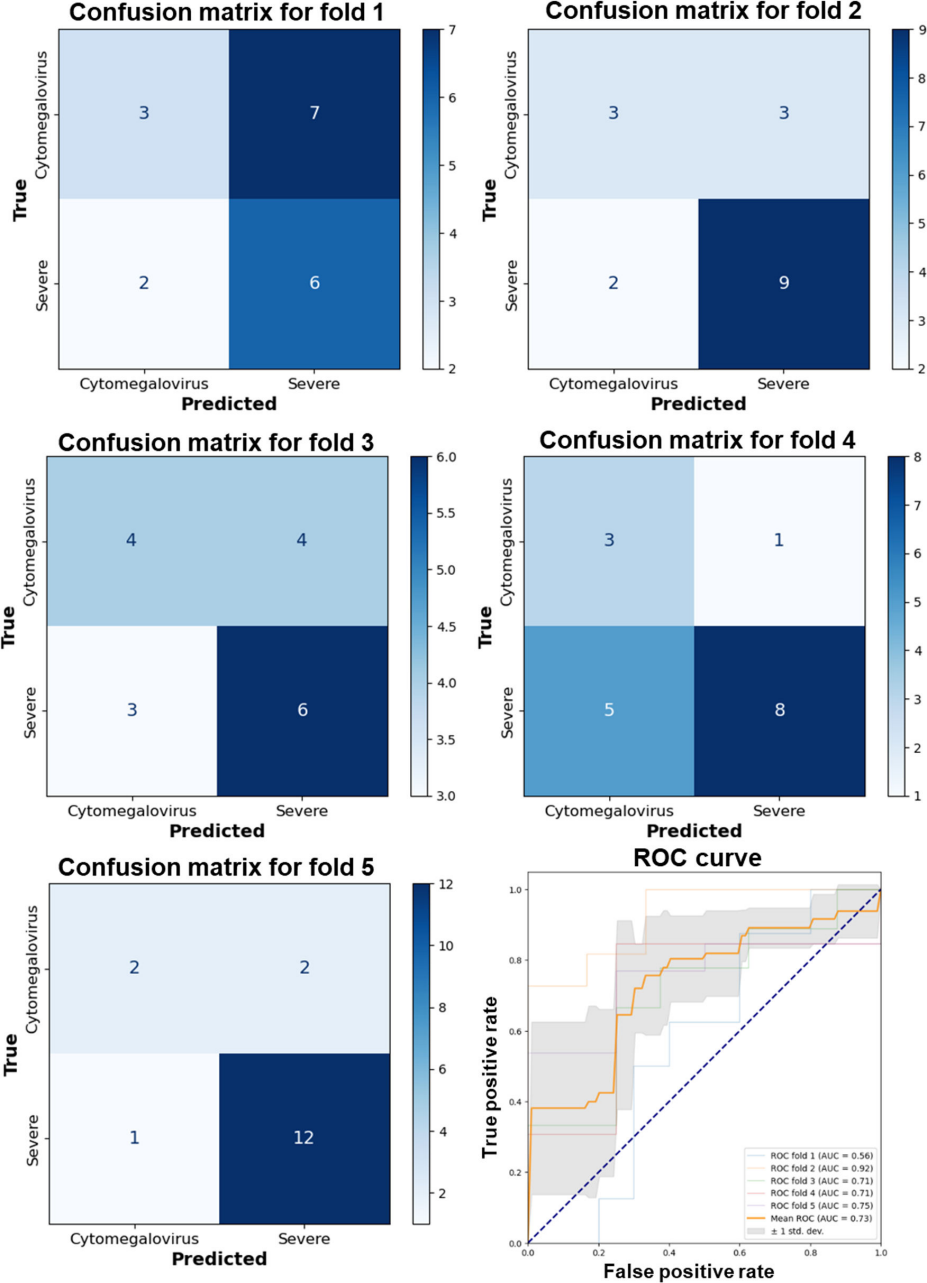
Performance evaluation of the VGG 19 model using 5-fold cross-validation and receiver operating characteristic curve analysis. AUC: area under the curve; ROC: receiver operating characteristic.

Further analysis using ROC curves and the AUC metric provided additional insights into model performance, demonstrating a long-term high true positive rate against a lower false positive rate, indicative of robust diagnostic capabilities. DenseNet 121 consistently exhibited an average AUC of 0.846 (SD 0.100) across folds (Figure 4). Meanwhile, EfficientNet B0 and B1 also displayed excellent classification abilities, with average AUCs of 0.796 (SD 0.098) and 0.838 (SD 0.107), respectively (Figures5  6). VGG 19, while presenting an average AUC of 0.730 (SD 0.115), showcased instances of high performance, particularly in folds where it achieved an AUC as high as 0.920 (Figure 7). However, a discrepancy was observed in the model’s performance through confusion matrix analysis; all models accurately identified severe UC conditions but exhibited inconsistency in identifying CMV conditions (Figures 4-7). This inconsistency was particularly evident for DenseNet 121 and EfficientNet B0, highlighting the need for improvement. These findings indicate that single models may encounter limitations when distinguishing CMV.

Incorporating the TTA technique into the ensemble model approach proved pivotal in addressing these classification challenges. The ensemble of 4 models augmented with TTA demonstrated remarkable efficacy in classifying endoscopic images of UC. The ensemble achieved an accuracy of 0.836 (95% CI 0.824‐0.848), a precision of 0.850 (95% CI 0.832-0.868), a recall of 0.904 (95% CI 0.890‐0.918), and an *F*_1_-score of 0.875 (95% CI 0.860‐0.890), indicating high reliability and balanced classification capabilities. Other ensemble configurations also yielded noteworthy results. The ensemble of 3 models with TTA exhibited precision of 0.814 (95% CI 0.801‐0.828), while the ensemble of 2 models with TTA demonstrated recall of 0.832 (95% CI 0.818‐0.846) and *F*_1_-score of 0.794 (95% CI 0.778‐0.810), indicating effective identification of true positive cases. Conversely, ensemble models without TTA showed a noticeable decline in performance metrics, with the ensemble of 4 models without TTA achieving an accuracy of 0.709 (95% CI 0.692‐0.726), a precision of 0.691 (95% CI 0.675‐0.707), a recall of 0.716 (95% CI 0.700‐0.732), and an *F*_1_-score of 0.752 (95% CI 0.737‐0.767). These results emphasize the significant role of TTA in enhancing classification performance and improving model generalization in distinguishing CMV from severe UC.

**Figure 8.**
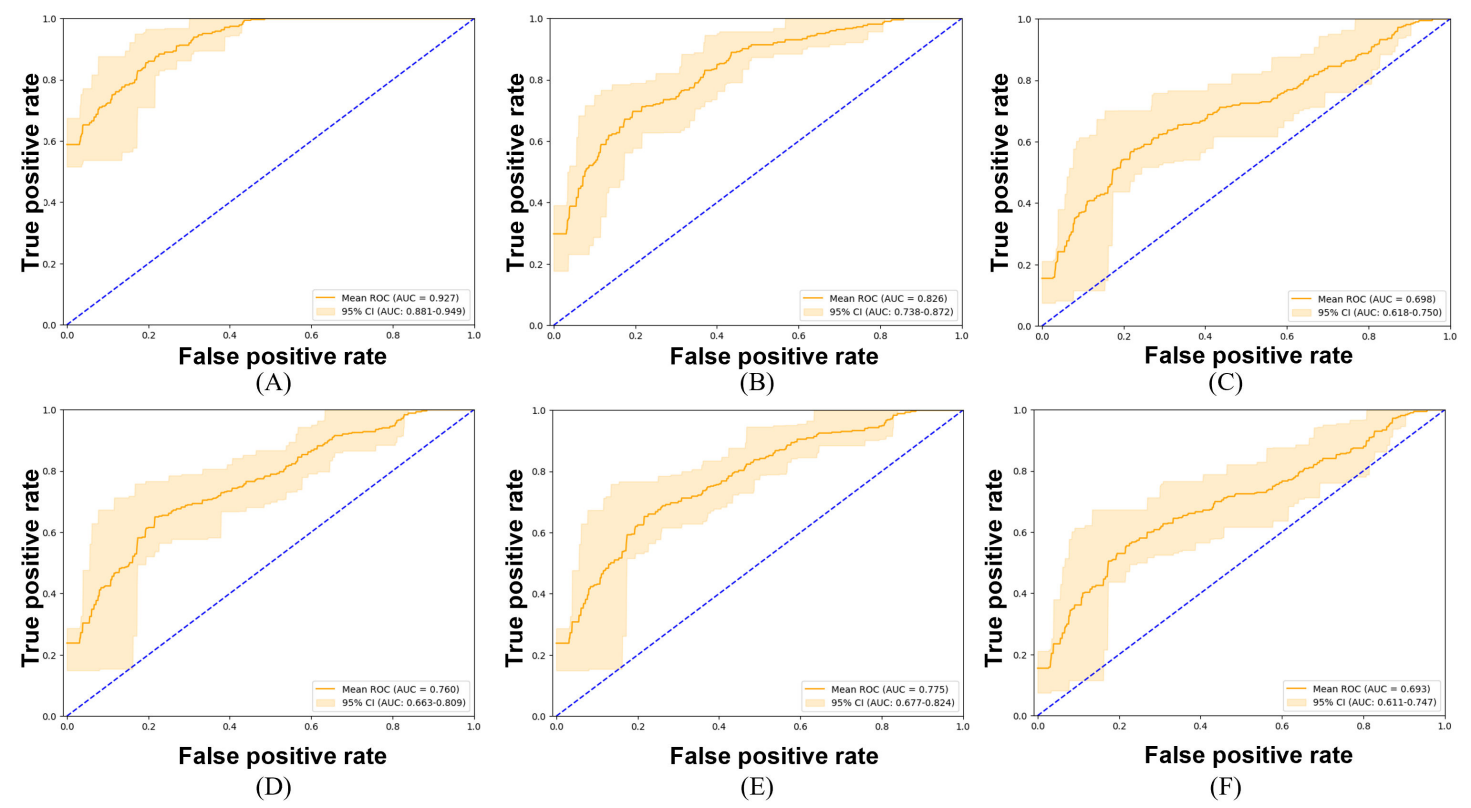
Receiver operating characteristic curves for endoscopic image classification models: (**A**) ensemble of 4 models with test-time augmentation, (**B**) ensemble of 3 models with test-time augmentation, (**C**) ensemble of 2 models with test-time augmentation, (**D**) ensemble of 4 models without test-time augmentation, (**E**) ensemble of 3 models without test-time augmentation, and (**F**) ensemble of 2 models without test-time augmentation. AUC: area under the curve; ROC: receiver operating characteristic.

The ROC curves reveal discernible patterns in model efficacy attributable to integrating TTA and the amalgamation of models within the ensemble (Figure 8). The ensemble of 4 models enhanced with TTA is particularly noteworthy, demonstrating the most pronounced diagnostic proficiency, with an AUC of 0.927 (95% CI 0.881‐0.949). This signifies superior diagnostic performance and a high true positive rate compared with the false positive rate. Despite a reduction in ensemble size, the ensemble comprising 3 models, with TTA implementation, maintains robust performance, as evidenced by an AUC of 0.826 (95% CI 0.738‐0.872). Conversely, the ensemble of 2 models with TTA displays a relatively inferior AUC of 0.698 (95% CI 0.618‐0.750).

In setups without TTA, there is generally a decrease in ensemble performance. The ensemble of 4 models without TTA achieves an AUC of 0.760 (95% CI 0.663‐0.809), confirming the efficacy of the ensemble approach even with a slight drop in effectiveness. Interestingly, a 3-model ensemble without TTA exhibits a slightly higher AUC of 0.775 (95% CI 0.677‐0.824), indicating a particularly effective combination of models. Conversely, the pair of models without TTA demonstrates the most notable decrease in performance, with an AUC of 0.693 (95% CI 0.611‐0.747), underscoring the significance of TTA in enhancing model performance and the benefits of larger ensembles for diagnostic accuracy.

## Discussion

### Principal Findings

The study underscores substantial advancements in medical diagnostics, particularly in gastroenterology, facilitated by applying deep learning technology. This research distinguishes itself by using an ensemble of deep learning models, including DenseNet121, EfficientNet B0, EfficientNet B1, and VGG 19, to differentiate between severe UC and CMV infection using endoscopic images. By leveraging an ensemble approach, this study not only improves classification performance but also demonstrates the clinical applicability of deep learning in distinguishing between these 2 conditions.

The evaluation of single models revealed their limitations in identifying CMV, highlighting the need for a more comprehensive approach. This insight underscores the utility of the TTA ensemble technique in discerning CMV infections and accurately classifying severe UC from endoscopic images. Furthermore, this approach indicates the potential to surmount the constraints of single-model methodologies and enhance diagnostic accuracy in complex medical image classification tasks. The ability to overcome the constraints of single-model methodologies and enhance diagnostic accuracy in complex medical image classification tasks is a crucial advancement in this field. These findings suggest that integrating deep learning into clinical workflows can significantly improve real-world diagnostic processes.

The ensemble of 4 models with TTA achieved remarkable performance in classifying endoscopic images of severe UC, with an accuracy of 0.836 (95% CI 0.824‐0.848), precision of 0.850 (95% CI 0.832‐0.868), recall of 0.904 (95% CI 0.890‐0.918), and *F*_1_-score of 0.875 (95% CI 0.860‐0.890). These results indicate high reliability and balanced performance, reinforcing the ensemble’s ability to deliver accurate and consistent diagnoses. The improved diagnostic accuracy can have a direct impact on clinical decision-making, reducing diagnostic errors and enabling timely intervention.

The distinction between CMV and severe UC through endoscopic images has been a topic in previous research [11-13]. Although specific endoscopic features can differentiate these 2 conditions, the differences are often subtle and require meticulous observation. Features, such as severe ulcers, punched-out ulcers, geographical ulcers, and spontaneous bleeding, can be crucial in differentiating certain diseases [12]. However, identifying these features through conventional observation methods is often challenging. Applying the ensemble model with TTA represents a substantial advancement in this context [39]. It stands out as the first initiative to harness the capabilities of deep learning or machine learning for this purpose. The insights derived from the AUC values and the corresponding ROC curves are pertinent. The ensemble method enhanced by TTA underscores the potential of deep learning in medical diagnostics. When integrated into clinical workflows, these advanced models have the potential to substantially enhance patient care by enabling more precise and accurate diagnoses, thereby laying the groundwork for improved patient outcomes and marking a noteworthy advancement in health care technology.

To further contextualize these advantages in clinical practice, deep learning models can contribute to reducing interobserver variability, which is a critical challenge in medical imaging. By providing consistent and objective assessments, AI-driven diagnostic tools can complement physician expertise, particularly in resource-limited settings where access to highly experienced endoscopists may be restricted. In addition, AI-powered systems can expedite the diagnostic process, enabling real-time or near-instantaneous assessments that facilitate prompt therapeutic interventions. These improvements translate into enhanced patient management, reduced hospital stays, and minimized complications associated with diagnostic delays.

Furthermore, integrating AI-based diagnostic models into real-time clinical decision support systems could significantly enhance their clinical utility. Future research should explore embedding these AI models into endoscopic software or electronic health record systems. This integration could provide real-time diagnostic support to gastroenterologists, improve the efficiency of the diagnostic process, and facilitate quicker treatment decisions. Ultimately, this could become a key factor in enhancing the overall quality of patient care.

A substantial limitation identified during the research process was the occurrence of false negatives in the validation phase [40]. These findings underscore the necessity for further adjustments to improve the model’s sensitivity, particularly in medical diagnostics, where distinguishing between similar conditions, such as CMV and UC, is critical. False negatives can cause delayed or inappropriate treatment, negatively impacting patient outcomes [41]. To address this, future studies should incorporate advanced augmentation techniques to enhance model sensitivity while maintaining specificity. In addition, exploring hybrid AI approaches that combine deep learning with rule-based algorithms could further refine diagnostic accuracy [42,43].

Another limitation is the lack of datasets; the data used in this study were limited in scope and quantity, thus constraining the generalizability and reliability of the model. As with other studies using proprietary datasets, our research also faces challenges in generalization, as the model was trained on a specific dataset. However, it is important to note that endoscopic imaging, compared with other imaging modalities, exhibits relatively minimal variation across different manufacturers due to its reliance on optic imaging rather than quantitative imaging [44]. This consistency helps maintain uniformity in image quality, which can positively impact AI model training and application.

Nevertheless, to address these generalization limitations, future research endeavors aim to mitigate these challenges by acquiring more diverse and extensive data through collaboration with multiple institutions. Multi-institutional data collection and incorporating various patient populations will enhance the model’s robustness and ensure its applicability across different clinical settings. Incorporating data gathered from various patient groups and regions will enhance the model’s generalization capability and facilitate the development of more reliable diagnostic tools [45]. Such collaborative efforts across multiple institutions are essential for augmenting the accuracy and reliability of the model.

This study uses deep learning technology to introduce a novel approach to medical diagnostics, particularly in gastroenterology. As the first to use deep or machine learning in distinguishing between severe UC and CMV, it heralds new possibilities in medical diagnostics. Implementing deep learning, notably the ensemble model with TTA, facilitates rapid, noninvasive, and remarkably accurate diagnostic techniques. These holds promise for substantially enhancing patient experience compared with conventional diagnostic approaches, resulting in time and resource savings and diminishing the necessity for unnecessary invasive procedures. Such methodologies alleviate patient discomfort and risk, expediting treatment decisions.

### Conclusion

This study marks a significant advancement in medical diagnostics by leveraging deep learning, specifically an ensemble model with TTA, to differentiate between severe UC and CMV infections using endoscopic images. While our findings demonstrated high accuracy, precision, and recall, the model’s reliance on a specific dataset may limit its generalizability, necessitating further validation in diverse clinical settings. By introducing a streamlined, noninvasive, and accurate approach, this research highlights the potential of AI-driven diagnostics in gastroenterology while underscoring the need for further refinement to enhance clinical applicability.

## Abbreviations

AI: artificial intelligence
AUC: area under the curve
CMV: cytomegalovirus
DenseNet: Densely Connected Convolutional Network
H&E: hematoxylin and eosin
HSV: hue, saturation, value
IHC: immunohistochemistry
PCR: polymerase chain reaction
ROC: receiver operating characteristic
TTA: test-time augmentation
UC: ulcerative colitis
VGG: Visual Geometry Group
